# Hepatitis A in Young Adults in the Golestan Province, Northeast of Iran

**DOI:** 10.4103/0974-777X.62862

**Published:** 2010

**Authors:** Moradi Abdolvahab, Khodabakhshi Behnaz, Besharat Sima, Teimoorian M

**Affiliations:** *Microbiology, Golestan Research Center of Gastroenterology and Hepatology, Golestan University of Medical Sciences, Gorgan, Iran*

Sir,

Hepatitis A virus (HAV) is a small RNA virus, the only member of hepatovirus species from the picorna virus family.[1] Various strains of this virus have been identified throughout the world, which have the same antigene, but only one genotype has been identified in humans.[2] The main transferring method is fecal–oral and from person to person in communities with lower socioeconomic levels. It can adapt to different environmental conditions well and may be transferred to humans by polluted water and rotten food, causing disease.[1]

In 0.01% of acute hepatitis A cases, fulminate hepatitis can occur with an estimated mortality rate of 60–80%. Regarding the fact that Iran is considered as one of the areas of high prevalence for this disease by the WHO[3] and confirmed by the studies in Tehran, Sistan-o-Balouchestan, Yazd and Sari,[4–7] this study was carried out to analyze the seroepidemiology of hepatitis A disease in 17-year-olds in the Golestan province, Northeast of Iran.

In this cross-sectional study, 461 blood samples were collected from 17-year-olds referred for mass vaccination of hepatitis B virus, Golestan province, Northeast of Iran. Samples were preserved at −20°C. HAV antibody was measured in them using a Dia.Pro. Diagnostic Bioprobes Srl. via Columella n° 31 – Milano - Italy through the enzyme-linked immunosorbent assay method. Data were collected by questionnaires, which contained demographic data of the subjects, and were entered into SPSS-13 software and analyzed by the Chi-square test. Among 461 participants, 211 were males (45.8%) and 232 (50.3%) lived in urban areas.

Most of them (268 cases) were living in families with six to 10 persons. Four hundred persons out of the whole studied cases (86.8%) were positive for HAV antibody, including 87.2% of males and 86.4% of females. Results of this study were in agreement with those of Zabol (8.6%), Yazd (89.5%), Sari (84.9%) and Sari (90.36%).[4–7] But, the result of a study in Tehran on cases of 6 months to 15-year olds showed that the incidence of HAV antibody was 22.3%.[1] Other studies in Brazil had reported the rate of HAV antibody in more than 80% of cases,[8] which are in agreement with our findings. Some other studies reported a higher rate of this antibody in Turkey and India.[9] This was reported to be 12.2% in Japan and 3.8% in people younger than 20 years, and the presence of HAV antibody has a significant relationship with the economic status and place of living.[10]

There was no significant relationship between gender and HAV antibody positivity. HAV antibody was positive in 87.5% of urban inhabitants and 86% in rural areas (*P* value>0.05).

No statistically significant difference was found between the number of family members, level of education, ethnicity, place of residence, level of education in parents and the presence of HAV antibody.

It can also be concluded that the seroepidemiology of HAV is high in our area and it should be considered that it is a preventable disease through providing healthy water and observing public and environmental health.
